# Lemur Tyrosine Kinases and Prostate Cancer: A Literature Review

**DOI:** 10.3390/ijms22115453

**Published:** 2021-05-21

**Authors:** Elena Ferrari, Valeria Naponelli, Saverio Bettuzzi

**Affiliations:** 1 Department of Medicine and Surgery, University of Parma, Via Gramsci, 14, 43126 Parma, Italy; valeria.naponelli@unipr.it (V.N.); saverio.bettuzzi@unipr.it (S.B.); 2 National Institute of Biostructure and Biosystems (INBB), Viale Medaglie d’Oro 305, 00136 Rome, Italy; 3 Centre for Molecular and Translational Oncology (COMT), University of Parma, Parco Area delle Scienze 11/a, 43124 Parma, Italy

**Keywords:** prostate cancer, lemur tyrosine kinase, androgen receptor, Akt signalling, MAPK signalling

## Abstract

The members of the Lemur Tyrosine Kinases (LMTK1-3) subfamily constitute a group of three membrane-anchored kinases. They are known to influence a wide variety of key cellular events, often affecting cell proliferation and apoptosis. They have been discovered to be involved in cancer, in that they impact various signalling pathways that influence cell proliferation, migration, and invasiveness. Notably, in the context of genome-wide association studies, one member of the LMTK family has been identified as a candidate gene which could contribute to the development of prostate cancer. In this review, of published literature, we present evidence on the role of LMTKs in human prostate cancer and model systems, focusing on the complex network of interacting partners involved in signalling cascades that are frequently activated in prostate cancer malignancy. We speculate that the modulators of LMTK enzyme expression and activity would be of high clinical relevance for the design of innovative prostate cancer treatment.

## 1. Introduction

Protein phosphorylation is one of the most common post-translational modifications. The dynamic process of phosphorylation is mediated by protein kinases, whose catalytic activity consists in the reversible phosphorylation of specific amino acid residues. This biochemical modification, where a phosphate group is donated by the high-energy ATP molecule, profoundly modifies the accepting protein substrate [1]. Such a modification introduces a charged, bulky, hydrophilic group (the phosphate) in the side chain of specific amino acids of the target protein, changing its conformation and interaction capability.

Together with the de-phosphorylation reactions, catalysed by specific phosphoprotein phosphatases, protein kinases contribute to many critical events in cell fate determination, cell cycle control, and signal transduction. Just as importantly, kinase deregulation is often associated with metabolic deregulation as well as several diseases. Therefore, over the last decades, protein kinases and phosphatases, both expressing a relevant regulatory potential in cell signalling, have become important targets for the pharmaceutical industry. Consistently, several kinase databases and resources provide up-to-date information on human kinome in physiological conditions and in association with diseases, as well as on the progress of kinome therapeutics [2].

Protein kinases have evolved to generate different kinase families, distinct in sequence, structure, and specificity [3,4]; the Tyrosine Kinase (TK) family stands out among them as a major research focus. In fact, members of the TK family, which exhibit a significant association with diseases, have been successfully targeted by chemical inhibitors. This is the case of Receptor Tyrosine Kinases (RTKs), regulatory signalling proteins governing cancer growth and metastasis. In physiological conditions, cell surface RTKs are activated when their extracellular domain is engaged with its ligand, typically a growth factor, thus inducing receptor dimerisation and tyrosine kinase activity [5,6,7]. RTKs auto-phosphorylate tyrosine residues in their cytosolic region, and engage many downstream signalling proteins, activating signal cascades that regulate cell replication, growth, differentiation, and survival [8]. Instead, in cancer cells, oncogenic alterations of RTKs (caused by mutations, genomic amplification, and chromosomal rearrangements) result in aberrant RTKs activation and downstream signal transduction, disrupting the balance between cell proliferation and cell death [6,8]. Since RTKs play crucial roles in cancer development, they represent molecular therapeutic targets for cancer treatment. In fact, many small molecule inhibitors have been developed for malignancies associated with mutated RTKs [7,8,9,10]. By targeting the ATP binding site (or an adjacent region), they specifically inhibit the tyrosine kinase function, prevent the phosphorylation of intracellular signal molecules, and block intracellular signal pathways that favour tumorigenesis. RTKs FDA-approved inhibitors, updated as of 18 August 2019, and their use in oncology has been accurately discussed by Pottier and co-workers [9]. Despite their success in cancer chemotherapeutics, acquisition of resistance to RTKs inhibitors inevitably develops. This is mainly attributed to RTK secondary mutations, which prevent the inhibitor from binding to the ATP pocket. To reduce drug resistance, recent approaches have been based on second- and third-generation RTKs inhibitors resistant to mutations, as well as on the combined use of synthetic with naturally derived RTKs inhibitors [7,11,12]. Another approach for targeting RTKs is based on monoclonal antibodies directed against the extracellular domains of the receptor, and aimed at interfering with RTK activation; their use has also been proposed in combination with tyrosine kinase inhibitors of the same RTK target [6,13].

Despite the importance of kinases in physiological conditions and their frequent mutation in disease, there is a substantial section of the human kinome that has remained little studied, up until now. This is what emerged from the NIH Illuminating the Druggable Genome (IDG) Program, aimed at identifying and providing information on proteins that are currently not properly studied within commonly drug-targeted protein families [14,15].

The members of the Lemur Tyrosine Kinases (LMTKs) subfamily, located at a recent branch of kinase evolution, are on the list of the protein targets identified by the IDG Program for additional research (last updated on 11 June 2019). The published research studies on Lemur Tyrosine Kinases (*n* = 53 articles can be found in PubMed) of the last decade have mostly been dedicated to cancer and neurodegeneration investigations.

LMTKs comprise a novel group of three membrane-anchored kinases that, through a complex interactome network, regulates a wide variety of key cellular events, often impacting cell proliferation and apoptosis [16]. Current functional data support the hypothesis that LMTKs may regulate endosomal cargo recycling and move steroid receptors from the endocytic recycling compartment to the nucleus, where they can regulate the transcription of specific receptor-activated genes [16]. Although solid experimental evidence has substantiated various molecular functions of LMTKs, this protein kinase subfamily remains incompletely characterised.

Not unexpectedly, LMTKs have been discovered to be involved in cancer, as they affect various signalling pathways that severely influence cell behaviour, proliferation, migration, and invasiveness. Genome-wide association studies have identified seven loci of the human genome that are linked to prostate cancer (PCa) risk [17]. Indeed, one of these contains a *LMTK* gene. PCa is one of the most common cancers in Western countries. The major challenge that researchers face today is to identify PCa biomarkers which may help clinicians for an early diagnosis and a better treatment. Among the most promising PCa biomarkers is the evaluation of the expression levels of genes involved in the growth and survival of PCa cells and, in this regard, LMTK is a promising candidate gene [18].

### Prostate Cancer

PCa represents the second most diagnosed cancer and the fifth most common cause of cancer death in men worldwide [19]. The incidence of PCa has grown significantly in the last decades due to increased diagnostic pressure and demographic ageing. The aetiology is complex, and the factors associated with PCa onset and progression are only partially determined. Many risk factors have been reported for this disease, including ethnicity, genetic predisposition, age, diet, family history, and lifestyle. The involvement of genetic factors is clearly shown by the higher incidence of PCa in men of African descent. They have the highest incidence and mortality rates, while Asian men have the lowest [20,21,22,23]. However, there are important differences in the specific mortality of individuals of the same ethnic group living in different countries and adopting different lifestyles; this shows that environmental factors play an important role in the onset of PCa [24].

PCa is a biologically heterogeneous disease [25,26]. It can develop into an aggressive form, which rapidly evolves to metastases, or remain in the body as an indolent form, which may remain undiagnosed for many years or even for the whole life of the patient. The most recommended treatment options are surgical removal of the gland, chemotherapy and/or radiotherapy. These therapeutic strategies are effective in the early phases of the pathology, before PCa becomes metastatic and turns into an aggressive and castration-resistant form (castration-resistant PCa, CRPC) [20]. In the latter case, cancer cells no longer respond to androgen deprivation therapy, a widely used therapy with the aim of slowing down the growth of PCa in its early phase of development [27]. Unfortunately, when PCa becomes aggressive or metastatic, this approach is ineffective and can be only envisaged as a palliative treatment.

Integrating molecular aspects of PCa into risk stratification remains a big challenge for clinical medicine. The ability to distinguish tumours with different clinical courses would both help to optimise the therapeutic strategy and allow for a reduction in overtreatment. Overtreatment is the unnecessary treatment of men with low-risk PCa that leads to a significant reduction in their quality of life because they do not need to be cured but could be treated with active surveillance. The identification of predictive genomic biomarkers is essential to achieving this objective. Recent research focuses on the clinical evaluation of genetic alterations associated with different outcomes of the disease [20,28,29,30,31,32,33,34,35,36,37,38,39]. To date, various chromosomal aberrations have been identified in PCa by novel approaches such as next generation sequencing [40,41,42,43,44]. Most patients present different profiles of alterations, involving more than 100 mutations in different genes, impacting PI3k/Akt/mTOR, MAPK and WNT signalling pathways, the cell cycle, and DNA repair [40].

Pursuing this line of research, here we present a narrative review of published literature on the role of LMTKs in PCa, with the aim of outlining the main research directions in this field of interest. Our analysis is complemented by an evaluation on the natural tyrosine kinase inhibitor Epigallocatechin-3-gallate and on the signalling pathways affected by this natural compound, with well-known anticancer and chemopreventive effects on prostate cancer cells.

## 2. LMTK Proteins

### 2.1. LMTKs and Cancer

The roles played by LMTKs in the cytosol and in the nuclear environment range from cell signalling and membrane trafficking to gene expression, explaining why their dysregulation can be associated with cancer [16,45]. A change in LMTK expression or protein level has been observed in different cancers, reflecting either positive or negative correlation dependent on cancer type. For example, LMTK2 protein level is high in colorectal and ovarian cancer, but is low in lymphoma, lung, testis, prostate, and renal cancer [45]. These findings suggest that LMTK level has the potential to be used as a diagnostic/prognostic biomarker. For instance, LMTK3 expression turned out to be a negative prognostic factor in patient with gastric cancer; its overexpression in bladder cancer was correlated with bladder cancer malignancy and predicted poor survival [46,47].

Many studies argue that LMTK proteins represent new potential targets for cancer therapy. Conti and co-workers showed that LMTK2 silencing sensitises immortalised epithelial and cancer cells to TNF-related apoptosis-inducing ligand (TRAIL) cytotoxicity, by regulating the levels of anti-apoptosis BCL2 family members [48]. Similarly, knockdown of LMTK2 inhibited the proliferation of colon cancer cells by inactivating nuclear factor-κB (NF-κB) [49], while LMTK2 silencing repressed the proliferation and invasion of hepatocellular carcinoma cells in vitro through the inhibition of Wnt/β-catenin signalling [50]. All these findings suggest that LMTK2 inhibition might represent a valuable approach in the treatment of specific cancers.

The search for LMTK proteins inhibitors is ongoing. An ATP-competitive, LMTK3 small-molecule inhibitor has been identified by a high-throughput screening of a library of kinase inhibitor-biased compounds against the recombinant form of the catalytic domain of LMTK3. Biochemical, biophysical, and cellular assays contributed to the identification and characterisation of this inhibitor. Inhibition of LMTK3 decreased the proliferation of different human cancer cell lines, with a concurrent increase in apoptosis in breast cancer cell lines [51]. These findings pave the way for further development and optimisation of LMTK3 inhibitors.

### 2.2. Catalytic Specificity of LMTKs

The three structurally and evolutionary related Lemur Tyrosine Kinases, namely LMTK1, LMTK2, and LMTK3, constitute a family of regulated specific kinases that performs a diversified set of cellular functions, mainly involved in cell signalling and membrane trafficking [16]. Sequence homology with the kinase domain of Receptor Tyrosine Kinases (RTKs) suggested a tyrosine kinase catalytic activity for LMTK1 [52]. On the contrary, in vitro assays showed that LMTK2 exhibited only serine/threonine kinase activity in autophosphorylation reactions or with added substrates [53], disclosing the phospho-acceptor capability of this enzyme, as well. By analogy, LMTK1 and LMTK3 are to be regarded as serine/threonine kinases. Recent efforts to decipher LMTK3 consensus phosphorylation motif by using a positional scanning peptide library revealed a consensus sequence with a strict requirement for Arg residues at positions −3 and/or −2, and allowed for the design of an optimal LMTK3 peptide substrate, Ser as the phospho-acceptor site [51]. The authors claim that a similar approach using a Tyr residue as a phospho-acceptor site would be worth exploring, as LMTK3 is predicted in silico to have dual phospho-specificity (serine/threonine and tyrosine). Because of these different findings, the name Lemur Tyrosine Kinase does not precisely reflect the kinase property of this family of proteins and might be substituted with Lemur Tail Kinase, as suggested by some authors [54].

To highlight the correlations between LMTKs, we extracted the information regarding their residues involved in ATP binding/orienting and in the phospho-transfer reaction, together with the different protein and gene names of the three LMTKs from the UniProt Knowledgebase (UniProtKB) database [55]; these core data are listed in Table 1.

### 2.3. Localisation, Membrane Topology and Structural Features of LMTKs

According to The Human Protein Atlas, LMTK2 is almost ubiquitously expressed in human tissues, with evidence at protein level, while LMTK1 is and LMTK3 are expressed in many tissues, with evidence at protein level only in some of them [56]. LMTK2 and LMTK3 are predominantly anchored to cytoplasmic endo-membranes, although they were also detected in the nucleus [57].

LMTK proteins share an overall structure made of a N-terminal signal peptide, a transmembrane domain comprising one or two transmembrane elements, a highly conserved kinase domain, and a long C-terminal tail, reminiscent of the long tail of Lemurs [16]. Originally, the determination of the membrane topology was performed with LMTK2. Briefly, in fluorescence protease protection assays and by using Human Embryonic Kidney cells (HEK293 cells) transfected with LMTK2 tagged with GFP at either the -NH_3_^+^ or -COO^−^ terminus, LMTK2 was proved to be an integral membrane protein in which both the amino and carboxyl termini are exposed to the cytoplasm. This topology also locates the kinase active site within the cytoplasm [16,58].

**Figure 1:**
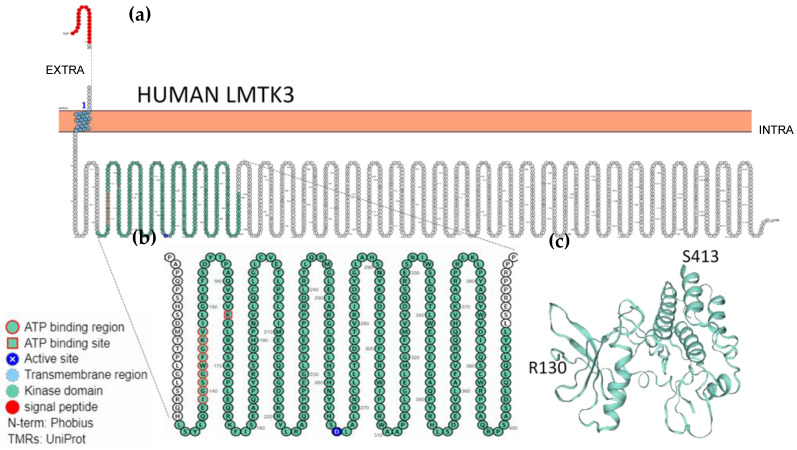
Sequence, predicted transmembrane topology, and kinase domain of human LMTK3. (**a**) Topology plot visualising the N-terminal signal peptide, the transmembrane region (blue), and the kinase domain (green), followed by the C-terminal tail domain. (**b**) An enlarged view of the catalytic kinase domain, evidencing the functional annotations associated with LMTK3_HUMAN UniProtKB entry and listed in the flanking legend. N-terminus location is predicted by Phobius server, while transmembrane regions (TMR) are derived from UniProt annotation. (**c**) Three-dimensional structure of the kinase domain, as displayed in SWISS MODEL Database (protein ID: 6seq.1).

Similar studies have not been performed with LMTK1 and LMTK3; therefore, their membrane topology can only be predicted on the basis of available data [55,59]. As an example, Figure 1 shows the protein sequence, the transmembrane topology, and the annotations of LMTK3 extracted from UniProtKB; data were processed using the Protter web-based tool (http://wlab.ethz.ch/protter) [59] to produce the topology plot and orientation of LMTK3 (Figure 1). Figure 1a,b highlight: 1. the transmembrane region with a unique transmembrane element; 2. the conserved kinase domain, comprising the ATP binding region, the ATP binding site, and the proton acceptor active site (Table 1); 3. the long C-terminal tail domain that constitutes the largest segment of the protein structure; 4. the cytoplasmatic localisation of both the kinase domain and the C-tail domain. The three-dimensional structure of the kinase domain of LMTK3 (Figure 1c) was retrieved from the SWISS MODEL Database (protein ID: 6seq.1). To date, this is the only domain of LMTKs whose structure has been solved (at 2.1-Å resolution), using X-ray diffraction and by studying a recombinant protein comprising the residues 134 to 444 [51]. The structure of the LMTK3 kinase domain shares structural and sequence similarities to different kinase domains, such as those of the transmembrane receptor epidermal growth factor receptor (EGFR), insulin receptor (INSR), and Janus kinase (JAK) [51]. This structural achievement is fundamental for further structural/functional studies and for facilitating the structure-based design of inhibitors that could help in the dissection of the signalling pathways in which LMTK3 is involved.

## 3. Literature Review

### 3.1. Evidence for LMTK2 Involvement in Prostate Cancer

Gene expression analysis was used to investigate the potential association of LMTK2 with the development of PCa in two different studies [60,61] (Table 2). The first study evaluated the expression of 7 PCa candidate genes in cancer versus benign prostatic hyperplasia (BPH) samples, in correlation with genotype at risk variants identified by genome-wide association studies (GWAS) [60]. The data confirm that the risk genotype at the GWAS variant rs6465657 correlates with LMTK2 expression and that prostate adenocarcinoma samples expresses 68% less LMTK2 mRNA than BPH samples, providing strong evidence for the role of this gene in PCa progression. The second study examined the expression level of LMTK2 in blood samples of prostate cancer patients, with the aim of making use of LMTK2 level as a potential biomarker for stratification between clinically insignificant and clinically significant PCa patients [61]. LMTK2 expression significantly decreases in blood samples of patients with PCa versus BPH as compared to control (with high PSA but no PCa), while PSA density value (ng/mL, based on PSA measurement divided by prostate volume) can differentiate PCa from benign prostate disease. The authors conclude that LMTK2 expression measurement, in conjunction with PSA density value, may assist in the identification of PCa clinical significance.

LMTK2 was reported to interact with different protein partners, originating from a complex interacting network that is relevant in human health and disease [16,45,54]. These studies provide details about LMTK2 interaction partners, as summarised below.

**Figure 2:**
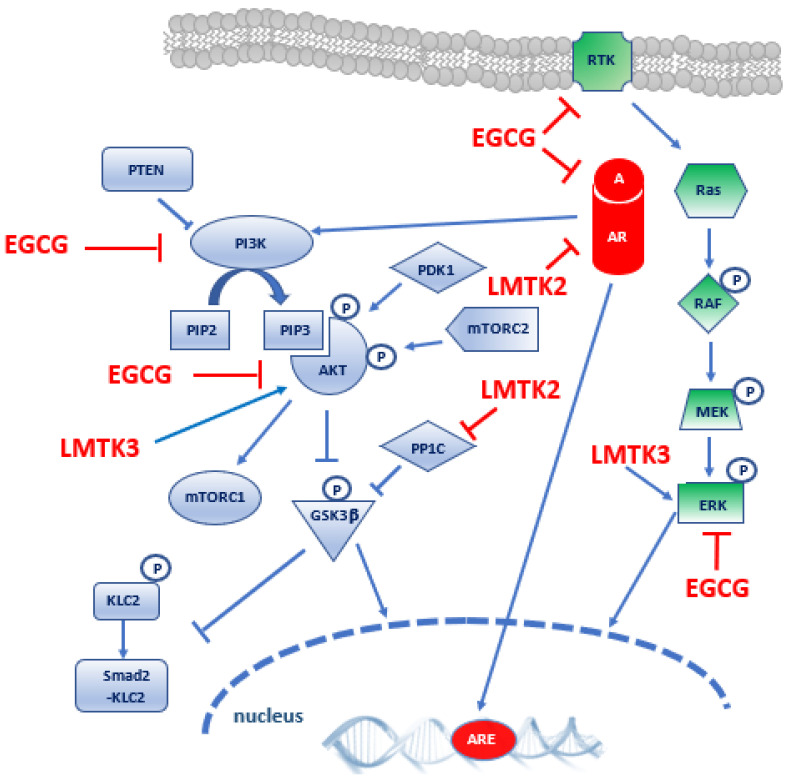
A simplified scheme of PI3K-Akt-mTOR signalling interaction with AR and RTK pathways. The image displays a model of PI3K-Akt-mTOR signalling via Class IA PI3Ks and crosstalk with AR and Ras/MAPK signalling cascades. Blue, red, and green objects represent PI3K-Akt-mTOR, AR and RTK signalling molecules, respectively. Inhibition arrows are red, activation arrows are blue, and the ℗ symbol indicates phosphorylation. Details on EGCG, LMTK2 and LMTK3 effects are provided in the text. A, androgen; Akt, protein kinase B; AR, androgen receptor; ARE, androgen responsive element; EGCG, epigallocatechin-3-gallate; ERK, extracellular signal regulated kinases 1 and 2; GSK3β, glycogen synthase kinase 3 beta; KLC2, Kinesin-1-Light Chain 2; MEK, mitogen-activated protein kinase kinase; mTOR, mammalian target of rapamycin; mTORC1/2, mTOR complex 1/2; PDK1, phosphoinositide dependent kinase 1; PI3K, phosphatidylinositol 3-kinase; PIP2, phosphatidylinositol 4,5-bisphosphate; PIP3, phosphatidylinositol (3,4,5)-trisphosphate; PTEN, Phosphatase and TENsin homolog; PP1C, protein phosphates 1C; RAF, rapidly accelerated fibrosarcoma; RAS homolog enriched in brain; RTK, receptor tyrosine kinase; Smad2, mothers against decapentaplegic homolog 2.

Androgen Receptor (AR) is a member of the steroid hormone nuclear receptor family and a ligand-dependent nuclear transcription factor that mediates the effects of androgens (Figure 2). AR controls prostate development and maintenance [62] and plays a key role in the development and progression of PCa [63,64]. As demonstrated by Shah and colleague (Table 2) [57], 1. LMTK2 and AR interact in prostate cancer epithelial cells and colocalise in human prostate tissue, and 2. in model cell lines, LMTK2 was discovered to be a negative regulator of AR transcriptional activity. In LNCaP cell line, LMTK2/AR complexes are predominantly extra-nuclear, but, in the presence of androgens, they localise in the nuclei. The study also shows that a decrease in LMTK2 expression is associated with increased risk of PCa and hyperplasia. Moreover, LMTK2 down-regulation obtained in PCa LNCaP cell line promotes tumour-forming capacity and proliferation, suggesting that the decrease in LMTK2 expression in PCa patients may promote tumour cells’ proliferation by enhancing AR transcriptional activity. Lastly, FKBP51, an AR-dependent gene and a positive regulator of AR activity, is highly expressed in castrate resistant PCa (CRPC) when compared with primary tumours. Accordingly, in LMTK2-KD PCa cells deprived of androgen FKBP51, expression becomes intensified, together with the mRNA levels of AR-dependent genes. This is a relevant piece of evidence that links LMTK2 to pathogenesis and progression of PCa to the castration-resistant phase.

Myosin VI is an actin-based motor protein involved in intracellular vesicular endocytic trafficking. Its expression is up-regulated in human PCa [65]. In LNCaP cells, in which myosin VI is also overexpressed, it is localised to early endosomes, recycling endosomes and trans-Golgi network (Table 2) [66]. It is also involved in the secretion of prostate-specific antigen (PSA) and vascular endothelial growth factor (VEGF), both supporting PCa growth. Interestingly, intracellular targeting experiments showed that Myosin VI interacts with, and coimmunoprecipitates with, LMTK2. Since both proteins participate in recycling endosome pathway and have a role in cancer progression, the authors emphasise the relevance of the secretory pathway by the recycling endosome to PCa pathogenesis.

Lastly, it has been recognised that LMTK2 also modulates the function of kinesin-1 molecular motor (Table 2) [67]. In fact, in HeLa cells, LMTK2 interacts with Protein Phosphatase-1C (PP1C) and induces its inhibitory phosphorylation, thus increasing inhibitory phosphorylation of the substrate Glycogen Synthase Kinase-3β (GSK3β). This inhibition 1. reduces Kinesin-1-Light Chain 2 (KLC2) phosphorylation, and 2. promotes KLC2 binding of Smad2 cargo and its transport to the nucleus mediated by Kinesin 1. This mechanism triggers the Smad2 signalling, induced by the Transforming Growth Factor-β (TGF-β) receptor stimulation. The reduction of LMTK2 expression by small interfering RNA (siRNA), mimicking the LMTK2 deficiency typical of PCa [57], reduced the binding of Smad2 to KLC2 and the downstream signalling. Therefore, since LMTK2 expression is significantly reduced in PCa tissues, Smad2 binding to KLC2 and transport on Kinesin-1 may also be inhibited in PCa cells.

### 3.2. Evidence for LMTK3 Involvement in Prostate Cancer

Two of the articles included in our review investigate the role of LMTK3 in PCa. The experiments of Sun and colleagues (Table 3) were performed in human PCa tissue as well as in two cell model systems: 1. PC3 and LNCaP cell lines; and 2. a transplant tumour model in nude mice, generated with PC3 cells infected with a recombinant lentivirus-LMTK3 construct [68]. The expression of LMTK3 appeared to be significantly reduced in cancer specimens compared to adjacent normal tissue. Instead, overexpression of LMTK3 in cancer cell lines decreased cell viability and migratory potential, also inducing apoptosis. In xenografts, this approach inhibited tumorigenicity of infected PC3 cells and induced apoptosis in vivo. Altered expression of apoptosis-related proteins Bcl-2, Bax and Caspase-3 in vitro and in vivo confirmed that the tumour suppression effect of LMTK-3 was mediated by modulating cell apoptosis. These findings suggest that LMTK3 may be a promising target for PCa therapy because of its potential and specific effect on PCa cell growth and apoptosis.

The three most important subfamilies of MAPKs (extracellular signal regulated kinases 1 and 2 [ERK1/2], c-Jun NH2-terminal kinase [JNK], and p38 mitogen-activated protein) and the cell signalling cascade of the phosphatidylinositol 3-kinase/protein kinase-B (PI3K/Akt) are known to play a key role in cell growth, differentiation, and apoptosis and can be activated in many different cancers. In accordance with this, the aforementioned data also demonstrate that, in PC3 cells and xenografts, the PI3K/Akt and MAPK signalling pathways may contribute to LMTK3 induced apoptosis. In fact, after LMTK3 overexpression, phosphorylation of Akt and ERK was inhibited, while phosphorylation and activation of p38 kinase and JNK were induced.

The rationale for these findings can be found in the coactivation of the RAS/MAPK and PI3K-Akt-mTOR signalling pathways, frequently occurring in human malignancies, including PCa (Figure 2). Indeed, a complex crosstalk between the two signalling cascades promotes PCa growth and metastasis [69].

Insulin-like growth factor 1 receptor (IGFIR) is a Receptor Tyrosine Kinase (RTK) driving cell proliferation, invasion, and survival. In the study of Gao and colleagues (Table 3), DU145 PCa cells were reverse-transfected using a kinase siRNA library and exposed to IGFIR AZ12253801 inhibitor to identify proteins that influence the viability response to the inhibitor [46]. LMTK3 depletion enhanced AZ12253801 sensitivity, suggesting that LMTK3 may be a candidate resistance mediator with roles in regulating receptor (or post receptor) signalling, thus confirming its role in the complex signalling cascade of IGFIR.

## 4. Epigallocatechin-3-Gallate

The overall incidence of PCa is lower in Asian countries compared to Western Countries, but the risk increases by nearly 20-fold for Asian immigrants living in the United States. This effect seems to be related to the adoption of a Western lifestyle, which contemplates lower intakes of vegetables, fruits, fish, tea, and soy, at the expense of increased consumption of meat and fatty food [71,72].

Natural bioactive compounds are molecules derived from plants and other natural sources which may exert health benefits. Bioactive compounds such as curcumin, lycopene, and green tea catechins have raised the interest of the scientific community because of their protective effect against cancer onset and progression [72,73,74]. Among them, Epigallocatechin-3-gallate (EGCG) is the main constituent of brewed green tea and polyphenol dried extract, as well as the most biologically active. Green tea extracts and its polyphenols, also known as catechins, are studied for their anti-cancer and chemopreventive effects on several cancer models, including PCa [75,76,77,78,79,80,81]. Multiple mechanisms mediate the effect of EGCG on PCa [82]. EGCG was shown to impact the same signalling pathways affected by LMTKs, such as PI3K/Akt/mTOR and MEK/ERK, as described in the “Discussion” section (Figure 2). In fact, EGCG is a competitive inhibitor of the class I PI3K and prevents the initial phosphorylation of Akt, necessary for the progression through the cell cycle. Moreover, EGCG inhibits mTOR and interferes with the signalling cascade downstream from mTOR, promoting the reactivation of apoptosis [83]. Furthermore, EGCG administration causes growth arrest and apoptosis thorough a decrease in ERK phosphorylation and an increase in JNK and p38 phosphorylation in human pancreatic cancer cells [84].

Moreover, EGCG induces the inactivation of AR-mediated transcription through its inhibitory action on histone acetyltransferase activity, which is necessary for the activation of AR transcription factors [85]. EGCG is also a direct small-molecule inhibitor of IGFIR activity in various cancer cell lines, resulting in the inhibition of cell proliferation and transformation [86].

The wide spectrum of effects exerted by EGCG is also mediated by its capacity to disrupt tightly packed and high-ordered membrane structures called lipid rafts that are involved in the regulation of cellular signalling. Lipid rafts are rich in RTKs, and these receptors are inhibited by EGCG in many cancer models [75].

## 5. Discussion

Despite the enormous progress made in the diagnosis and treatment of PCa, this pathology continues to be not only the second most diagnosed oncologic disease, but also one of the leading causes of cancer death in men worldwide. Most PCas require androgens to grow, which is why androgenic deprivation (ADT) is used as standard therapy to rescue the patient. ADT leads to the temporary inhibition of AR. Activated AR is a transcription factor of paramount importance for the regulation of cell growth, differentiation, migration, and survival in all androgen-dependent tissues. In prostate tissue, activated AR modulates the development of the gland, tissue homeostasis, and cell transformation. However, too often the clinical response to ADT is only temporary, since patients almost inevitably develop resistance to therapy over time. At that point, ADT becomes only palliative, though it still ameliorates the quality of life of patients. Because of the genetic and phenotypic heterogeneity of PCa, it is now very difficult to distinguish indolent tumours from aggressive ones. This makes prognosis, and personal treatment, rather difficult to achieve. The challenge that scientists face today is that of achieving a better risk stratification of PCa to reduce under-diagnosis, unnecessary biopsy, or overtreatment in order to enable clinicians to identify the optimal personalised therapeutic plan for each patient. Scientific research has long been committed to the development of a solid predictive genetic signature for better stratification of patients diagnosed with PCa [87,88,89], but more work needs to be done. Nevertheless, the advent of GWAS studies has given new impetus to the investigations on PCa carcinogenesis and the potential involvement of specific genes in the process of prostate cell transformation.

As evidenced in the present review, LMTK2 and LMTK3 are involved in PCa development. Their level of expression appears to be reduced in human prostate tissue as compared to normal tissue [57,60,68]. Additionally, LMTK2 blood level has been shown to contribute to the identification of clinically significant PCa [61]. We report that both LMTK2 and LMTK3 interact with, and modulate, the activity of specific enzymes of the signalling cascades regulating cell growth, differentiation, and apoptosis in PCa cells. LMTK interacting partners are involved in PI3K/Akt and Ras/MAPK signalling cascades, which are frequently coactivated in human malignancies, including PCa and CRPC [69]. 

In PCa cell lines, LMTK3 overexpression increased the levels of phosphorylated p38 and JNK, two MAPKs that mediate apoptotic death, and decreased ERK1/2 and Akt phosphorylation [68]. In this way, LMTK3 acts as a tumour suppressor that regulates cell apoptosis, modulating the level of anti-apoptotic Bcl-2 and decreasing pro-apoptotic Bax and caspase-3. Therefore, the low levels of LMTK3 found in PCa tissue may be associated with a decreased apoptotic rate of cancer cells.

A direct interaction between AR and LMTK2 and its involvement in the translocation of AR to the nucleus has been speculated after the discovery of the LMTK2-AR complex localisation in the nuclear fraction of PCa cells [64]. Notably, AR signalling axis converges with RAS/MAPK and PI3K-Akt-mTOR to promote PCa development (Figure 2); in fact, AR signalling regulates cell growth, differentiation, and survival, thus playing a critical role as a transcriptional regulator in prostate tissue homeostasis and in PCa [69], while PI3K/Akt/mTOR and Ras/MAPK signalling favours epithelial–mesenchymal transition and cell migration/invasion [90].

Since PI3K-Akt-mTOR pathway appears to be frequently activated in many aggressive PCa, targeting this pathway constitutes a valuable opportunity in PCa treatment. A better understanding of the dynamics of the three interacting signalling pathways is needed to design new therapeutic approaches based on combining the inhibition of PI3K/Akt/mTOR pathway with the inhibition of either the Ras/MAPK or AR signalling pathways [69,91].

In this scenario, LMTKs represent valid and promising therapeutic targets in PCa management, since their overexpression is expected to modify the complex signalling network involving PI3K/Akt/mTOR, RAS/MAPK and AR pathways. Despite the large number of compounds tested in vitro as specific inhibitors able to target different members of these signalling cascades, many of them did not reach the phase II of clinical trials. The reason for this failure can be found in the intense relationships and crosstalk between these signalling pathways, allowing for a compensatory response to the candidate drug effect, thus making the treatment ineffective [92].

Since PCa treated with ADT eventually switch to an androgen refractory state, where cells express survival genes bypassing hormone-dependent AR recruitment, myosin VI has been suggested as a potential therapeutic target for PCa, given that it acts as a regulator element able to interact with AR and affect AR-dependent protein levels. In this regard, LMTK2 is known to bind directly to myosin VI in human cervical cancer HeLa cells, permitting the delivery of membrane pumps, channels, and nutrient receptors from the early endosome to the perinuclear endocytic recycling compartment (ERC) and to the plasma membrane [93]. Moreover, LMTK2 has been discovered to be related to the secretory pathway via the recycling endosome and to Smad2 transcription factor transport to the nucleus following TGF-β stimulation. This is demonstrated by the interaction of LMTK2 with two different molecular motors, myosin VI and kinesin-1, respectively [66,67]. Moreover, given these findings, we predict that the modulation of LMTK2 levels or activity may also have an important impact on these two molecular motors with expected important alterations in PCa cells.

The development and characterisation of specific modulators of LMTKs expression is a promising field of research. Interestingly, the treatment with the synthetic Protein Kinase C (PKC) activator tetradecanoylphorbol-13-acetate (TPA) in mouse embryonic fibroblast cells (NIH 3T3) increases the protein levels of LMTK2 through the binding of activator protein-1 (AP-1) transcription factor complex to LMTK2 promoter and the consequent increase of mRNA production and LMTK2 synthesis [94]. The coadministration of TPA and EGCG in cancer model systems might be a promising approach to promote both the synergic inhibition of AR signalling mediated by the LMTK2 inhibitory action and the down regulation of PI3K-Akt-mTOR pathway directly by means of EGCG. 

In conclusion, the identification of regulators of expression and/or activity of LMTKs are of high clinical relevance for the design of novel PCa treatment strategies. We speculate that such LMTKs effectors might exert a synergistic antitumor action with EGCG, a natural plant-derived polyphenolic compound with known chemopreventive and anticancer effects on prostate cancer cells.

## 6. Methods: Search Strategy and Study Selection

The studies included in this review were research articles reporting on LMTK involvement in PCa. References were obtained by searching Pubmed, Scopus, and WoS databases. In all the databases considered, the search term Lemur Tyrosine Kinase was used alone and in association with PCa, using and as Boolean operator to combine subjects. The LMTK acronym was also used along with the numbers 1, 2, and 3, to specify the three structurally and evolutionary related members of this protein kinase family.

The publication date range considered for study selection was January 2005–January 2020, although a literature search performed in March 2021 added a supplemental study [61].

We imported the results of each search into the EndNote^®^ software (Clarivate Analytics) for reference management, merging all records in a single collection. After the elimination of duplicates, two reviewers with biochemical backgrounds performed the eligibility assessment in parallel, without final disagreement. The final collection consisted of seven research articles.

The included studies were obtained as full-text articles and thoroughly examined. For each study, the information relevant to the present revision was extracted and documented in especially designed spreadsheets (Table 2 and Table 3), under the following headers: Reference, Cell line/tissue, Principal techniques, Main results, and Conclusions.

## Figures and Tables

**Table 1:** LMTKs core data ^1^ extracted from the UniProt Knowledgebase database.

Entry Name	Protein Names	Gene Names	Length (Res.)	Proton Acceptor Active Site	ATP Binding Site	ATP Nucleotide Binding (Res. Number)
LMTK1_HUMAN	Serine/threonine-protein kinase LMTK1, (EC 2.7.11.1) (Apoptosis-associated tyrosine kinase) (AATYK) (Brain apoptosis-associated tyrosine kinase) (CDK5-binding protein) (Lemur tyrosine kinase 1) (p35-binding protein) (p35BP)	AATK, AATYK, KIAA0641, LMR1, LMTK1	1374	D253	K156	131–139
LMTK2_HUMAN	Serine/threonine-protein kinase LMTK2, (EC 2.7.11.1) (Apoptosis-associated tyrosine kinase 2) (Brain-enriched kinase) (hBREK) (CDK5/p35-regulated kinase) (CPRK) (Kinase/phosphatase/inhibitor 2) (Lemur tyrosine kinase 2) (Serine/threonine-protein kinase KPI-2)	LMTK2, AATYK2, BREK, KIAA1079, KPI2, LMR2	1503	D265	K168	143–151
LMTK3_HUMAN	Serine/threonine-protein kinase LMTK3 (EC 2.7.11.1) (Lemur tyrosine kinase 3)	LMTK3, KIAA1883, TYKLM3	1460	D266	K164	139–147

^1^ Data were obtained by entering the search terms “Human Lemur Tyrosine Kinase” and filtering by those that were reviewed, i.e., by selecting the records with information extracted from the literature and curator-evaluated computational analysis.

**Table 2:** Evidence from literature for LMTK2 involvement in prostate cancer.

Reference	Cell Line/Tissue	Principal Techniques	Main Results	Conclusions
Harries et al. [60]	Human prostate samples (cancer and benign prostatic hyperplasia, BPH) from Exeter tissue bank	PCR amplification and sequencing for genotyping 7 GWAS risk loci	Risk genotype at the GWAS variant rs6465657 correlates with LMTK2 expression	Expression levels of LMTK2 inversely correlate with the presence of prostate cancer
		Real-time PCR	Prostate adenocarcinoma samples expressed 68% less LMTK2 mRNA than BPH samples	
Vezelis et al. [61]	Blood sample of patients who had rising PSA after negative transrectal systematic prostate biopsy	Analysis of CRISP3, LMTK2 and MSMB gene expression by means of quantitative RT-PCR	LMTK2 and MSMB expression significantly decreases in blood samples of patients with PCa and Benign Prostate Disease as compared to control PSA density (ng/mL) can differentiate PCa from the benign prostate disease	PSA density, in combination with LMTK2 expression level, may assist in stratification between clinically insignificant and clinically significant PCa
Shah et al. [57]	Human prostate tissue array (prostate cancer, hyperplasia, and normal prostate tissue)	Immunostaining analysis	LMTK2 is down regulated in human PCa	Loss of LMTK2 protein is strongly associated with prostate cancer and prostate hyperplasia LMTK2 interacts directly with AR and inhibits its transcriptional activity LMTK2 down-regulation promotes tumour forming capacity and proliferation The decrease in LMTK2 expression in prostate cancer patient may promote tumour cells proliferation by enhancing AR transcriptional activity
	LNCaP cells Normal human prostate tissue	Coimmunoprecipitation Colocalisation analysis by immunostaining	LMTK2 and AR interact in prostate cancer cells and colocalise in human prostate tissue	
	HEK293 cells	Dual luciferase assay with *LMTK2* knockdown or *LMTK2* overexpression	Knockdown of *LMTK2* in cells expressing AR enhances androgen-dependent activation of a luciferase reporter gene Overexpression of *LMTK2* in cells expressing AR decreases androgen-dependent activation of a luciferase reporter gene	
	LNCaP cells	Real-time PCR	*LMTK2* knockdown cells, deprived from androgens, show a significant increase in mRNA expression of AR responsive genes	
	LNCaP cells	Tumoursphere assay Cell viability assay	LNCaP knockdown cells: -showed higher colony-forming capacity -showed ~5 times higher cell viability under androgen starvation	
Puri et al. [66]	LNCaP cells	Immunofluorescence microscopy	Myosin VI is present on early endosomes, recycling endosomes and trans-Golgi network	LMTK2, together with Myosin VI, may participate in the orchestration of endosomal recycling pathway The secretory pathway via the recycling endosome can be involved in PCa pathology
		Coimmunoprecipitation	LMTK2 binds to and coimmunoprecipitates with Myosin VI	
		Myosin VI siRNA knockdown	Secretion of PSA and VEGF is reduced	
Manser et al. [67]	HeLa cells	Coimmunoprecipitation Immunoblot analysis	LMTK2 interacts with Protein Phosphatase-1C LMTK2 increases inhibitory phosphorylation of Glycogen Synthase Kinase-3β LMTK2 reduces Kinesin-1-Light Chain 2 phosphorylation and promotes KLC2 binding of Smad2 transcription factor	Since LMTK2 expression is significantly reduced in prostate cancer tissues, Smad2 binding to KLC2 and transport on Kinesin-1 may also be inhibited in prostate cancer cells
		siRNA knockdown of LMTK2	LMTK2 knockdown inhibits Smad2 nuclear signalling in response to TGF-β receptor activation	

AR, androgen receptor; PSA, prostate-specific antigene; VEGF, vascular endothelial growth factor; TGF-β, Transforming Growth Factor− β; GWAS, genome wide association studies (for prostate cancer); CRISP3, cysteine rich secretory protein 3; MSMB, microseminoprotein beta.

**Table 3:** Evidence from the literature for LMTK3 involvement in prostate cancer.

Reference	Cell Line/Tissue	Principal Techniques	Main Results	Conclusions
Sun et al. [68]	Prostate cancer tissue	Quantitative RT-PCR Immunoblot analysis data	Expression of LMTK3 is reduced as compared to normal tissue	A low level of LMTK3 expression is associated with PCa LMTK3 overexpression can induce PCa apoptosis in vitro and in vivo, and Akt and MAPK signalling pathways may contribute to this process Low levels of LMTK3 ex-pression in PCa tissue may reflect a decreased apoptotic rate
	PC3 and LNCaP prostate cancer cells infected with recombinant lentivirus-*LMTK3*	MTT and TUNEL assays Transwell and Matrigel invasion assays Immunoblot analysis	Overexpression of LMTK3: − decreases cell viability and induces apoptosis − attenuates cell invasion and migration ability − inhibits phosphorylation of Akt and ERK, and promotes phosphorylation of p38 kinase and JNK in PC3 cells	
	Subcutaneous tumour model in nude mice, based on PC3 cells infected with recombinant lentivirus-*LMTK3*	Caliper measurement TUNEL assay Immunoblot analysis	Overexpression of LMTK3: − decreases tumour volume − increases % of apoptotic cells − inhibits phosphorylation of Akt and ERK; and promotes phosphorylation of p38 kinase and JNK in xenografts	
Gao et al. [70]	DU145 prostate cancer cells	Reverse transfection with a kinase siRNA library Exposition to IGFIR1 AZ12253801 inhibitor	AZ12253801 inhibits IGFIR phosphorylation and cell viability LMTK3 depletion enhances AZ12253801 sensitivity	LMTK3 is among the putative mediators of resistance to IGFIR inhibition

IGFIR, insulin-like growth factor I receptor (receptor tyrosine kinase).
